# The effect of water immersion on short-latency somatosensory evoked potentials in human

**DOI:** 10.1186/1471-2202-13-13

**Published:** 2012-01-24

**Authors:** Daisuke Sato, Koya Yamashiro, Hideaki Onishi, Yoshimitsu Shimoyama, Takuya Yoshida, Atsuo Maruyama

**Affiliations:** 1 Institute for Human Movement and Medical Sciences, Niigata University of Health and Welfare, Shimami- cho 1398, kita-ku, Niigata city, Japan, 950-3198; 2 Department of Health and Sports, Niigata University of Health and Welfare, Shimami- cho 1398, kita-ku, Niigata city, Japan, 950-3198; 3 Department of Physical Therapy, Niigata University of Health and Welfare, Shimami- cho 1398, kita-ku, Niigata city, Japan, 950-3198

## Abstract

**Background:**

Water immersion therapy is used to treat a variety of cardiovascular, respiratory, and orthopedic conditions. It can also benefit some neurological patients, although little is known about the effects of water immersion on neural activity, including somatosensory processing. To this end, we examined the effect of water immersion on short-latency somatosensory evoked potentials (SEPs) elicited by median nerve stimuli. Short-latency SEP recordings were obtained for ten healthy male volunteers at rest in or out of water at 30°C. Recordings were obtained from nine scalp electrodes according to the 10-20 system. The right median nerve at the wrist was electrically stimulated with the stimulus duration of 0.2 ms at 3 Hz. The intensity of the stimulus was fixed at approximately three times the sensory threshold.

**Results:**

Water immersion significantly reduced the amplitudes of the short-latency SEP components P25 and P45 measured from electrodes over the parietal region and the P45 measured by central region.

**Conclusions:**

Water immersion reduced short-latency SEP components known to originate in several cortical areas. Attenuation of short-latency SEPs suggests that water immersion influences the cortical processing of somatosensory inputs. Modulation of cortical processing may contribute to the beneficial effects of aquatic therapy.

**Trial Registration:**

UMIN-CTR (UMIN000006492)

## Background

Water immersion activates several distinct somatosensory modalities, including tactile, pressure, and thermal sensations. Somatosensory inputs can induce a variety of cardiovascular and respiratory responses, including decreased heart rate, increased stroke volume [1], and reduced functional residual capacity [2]. These physiological responses can have therapeutic benefits; indeed, water immersion is used as part of rehabilitation regimes for orthopedic, cardiovascular, and respiratory disorders. Water immersion once a week also improved the activities of daily living (ADL) in some frail elderly and hemiplegic patients after stroke [3]. Benefits to neurological patients suggest that water immersion may influence cerebrocortical processing, but this remains to be determined. Elucidating the cortical somatosensory processes induced by water immersion and the effects of water immersion on the processing of other sensory inputs will help delineate the mechanisms of sensory integration and could facilitate the development of improved aquatic therapies for neurology patients.

Somatosensory input from peripheral nerves activates several cortical areas. This modulation of somatosensory input can be evaluated by somatosensory-evoked potentials (SEPs). SEPs are divided into short-latency and long latency types. Short-latency SEPs (with latencies of 20 to 40 ms) are generated in area 3b and/or area 1 during thalamocortical inputs [4] and reflect the first stage of cortical somatosensory processing. Many reports have shown that somatosensory input attenuates short-latency cortical SEPs evoked by median nerve stimuli in the cerebral cortex or in the subcortical structures during movement [5,6] and in the presence of interfering tactile stimuli [7,8]. In 1981, Jones et al. [9] investigated whether short-latency SEPs evoked by median nerve stimuli were affected by various interfering tactile stimuli, and proposed that short-latency SEPs could be influenced by light touch stimuli of the body. In scalp recordings, continuous interfering tactile stimuli applied to the median nerve distribution of the hand primarily affected early SEP components peaking at about 20-30 ms, inverting the polarity between the parietal and frontal regions [7,10]. Subsequently, Jones et al. [8] showed that short-latency SEPs generated in somatosensory cortical areas 3b and 1 were attenuated by interfering tactile stimuli and cutaneous pressure [8]. Based on these studies, it appears that somatosensory inputs evoked by water immersion, such as the tactile sense of water and hydrostatic pressure, may affect the short-latency SEPs generated in areas 3b and 1.

In our previous study using functional near infrared spectroscopy (fNIRS), water immersion was associated with increased oxygenated hemoglobin (oxyHb) concentrations within the sensorimotor area of the cortex, including areas 3b and 1. This finding suggests that the somatosensory input from water immersion might activate this cortical area [11]. However, to the best of our knowledge, there is no direct evidence that water immersion has any effect on somatosensory cortical processing. In the present study, we examined the effect of water immersion on the short-latency SEPs in response to median nerve stimuli. Based on the aforementioned results, we hypothesized that somatosensory inputs from water would attenuate these short-latency SEPs.

## Results

**Figure 1:**
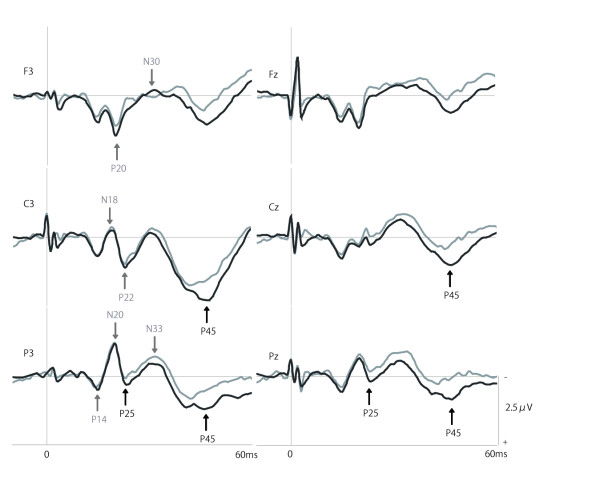
**Grand averaged short-latency components of the SEP waveforms from each electrode**. P20, N30, and P45 were measured by electrodes F3 and Fz (top panel), N18, P22, N30, and P45 by electrodes C3 and Cz (middle panel), and N20, P25, N33, and P45 by electrodes P3 and Pz (lower panel). Black lines are the grand averaged waveforms under nonimmersed control conditions from the 10 subjects. Grey lines are the grand averaged waveforms during water immersion.

Figure 1 presents the grand average SEP waveforms from all ten subjects. We measured the amplitudes of P20, N30, and P45 from electrodes F3 and Fz, N18, P22, N30, and P45 from electrodes C3 and Cz, and N20, P25, N33, and P45 from electrodes P3 and Pz. Each SEP component was identified by unique latency, polarity, and scalp distribution and each was consistently recorded in all subjects.

Table 1 shows the amplitudes and latencies of these SEP components under nonimmersed control and immersed conditions. Results of a two-factor repeated measures ANOVA revealed no significant interaction between conditions (control or immersed) and the specific electrode on the amplitude of these SEP components. There was a significant main effect of condition in the parietal region (P25: F(1,9) = 16.03, P < 0.01; P45: F(1,9) = 10.21, P < 0.05) and in the central region (P45: F(1,9) = 12.51, P < 0.01). Tukey's post hoc tests revealed significant effects of immersion on the P45 amplitude measured by the central electrodes C3 and Cz, and on P25 and P45 amplitudes measured by the parietal electrodes P3 and Pz. In contrast, the P20, N30, and P45 amplitudes measured by the F3 and Fz electrodes were not significantly different between nonimmersed control and immersed conditions. Similarly, the N18, P22, and N30 amplitudes measured by the C3 and Cz electrodes were not significantly different between the nonimmersed control and immersed conditions. Finally, the N20 and N33 amplitudes measured by P3 and Pz electrodes were not significantly altered by water immersion. Results of the two-factor repeated measures ANOVA revealed no significant interaction between condition and electrode on the latency of any SEP component, and no significant main effect of experimental condition on the all electrodes.

**Table 1:** Mean amplitudes and latencies of the SEP components (SE)

		amplitude(μV)				latency (ms)			
		
Electrode	Component	Nonimmersed		Immersed		Nonimmersed		Immersed	
Fz	P20	0.90	(0.12)	0.89	(0.12)	20.30	(0.54)	20.60	(0.62)
	N30	-2.21	(0.38)	-2.27	(0.36)	32.20	(1.61)	32.20	(1.73)
	P45	1.85	(0.33)	1.58	(0.26)	47.90	(0.66)	47.30	(0.86)
F3	P20	1.09	(0.16)	0.98	(0.13)	20.40	(0.22)	20.50	(0.27)
	N30	-2.17	(0.39)	-1.92	(0.35)	30.90	(1.18)	30.20	(1.49)
	P45	2.09	(0.40)	1.67	(0.31)	46.40	(1.36)	46.00	(1.05)
Cz	N18	-0.79	(0.17)	-0.97	(0.27)	18.60	(0.60)	19.10	(0.84)
	P22	0.62	(0.10)	0.70	(0.20)	23.00	(0.99)	22.70	(1.07)
	N30	-1.49	(0.27)	-1.52	(0.26)	32.20	(0.93)	32.30	(0.78)
	P45	2.16	(0.23)	1.70	(0.19)	47.00	(1.04)	45.50	(0.86)
C3	N18	-1.29	(0.16)	-1.24	(0.18)	18.80	(0.29)	18.70	(0.30)
	P22	2.11	(0.32)	1.99	(0.29)	23.60	(0.54)	23.30	(0.50)
	N30	-1.88	(0.38)	-1.94	(0.38)	30.70	(0.33)	30.80	(0.47)
	P45	2.90	(0.31)	2.68	(0.31)	46.70	(1.30)	45.00	(1.30)
Pz	N20	-1.33	(0.14)	-1.42	(0.12)	19.60	(0.34)	20.25	(0.35)
	P25	1.23	(0.20)	1.02	(0.21)	23.80	(0.49)	24.25	(0.60)
	N33	-1.20	(0.14)	-1.19	(0.11)	31.20	(0.59)	30.88	(0.68)
	P45	1.52	(0.18)	1.36	(0.19)	43.70	(1.34)	41.88	(0.86)
P3	N20	-1.96	(0.23)	-1.98	(0.24)	19.70	(0.21)	19.70	(0.15)
	P25	2.13	(0.31)	1.83	(0.34)	24.40	(0.70)	24.20	(0.63)
	N33	-1.33	(0.19)	-1.22	(0.18)	31.40	(0.64)	31.50	(0.60)
	P45	1.92	(0.22)	1.74	(0.24)	42.20	(1.01)	41.10	(0.77)

## Discussion

Water immersion has been shown to benefit many patients, including patients recovering from stroke, suggesting that this form of therapy might alter cortical activity. In the present study, such an effect was directly demonstrated under controlled experimental conditions. Water immersion significantly attenuated short-latency SEPs evoked by median nerve stimuli. While the amplitudes of the central N18, P22, and N30, the frontal P20 and N30, and the parietal N20 and P33 components were not changed significantly by water immersion, the parietal P25 and P45 amplitudes as well as the central P45 amplitude were significantly smaller under the immersed condition. These results suggest that water immersion has a gating effect on short-latency SEPs evoked by median nerve stimuli, and can therefore influence the cortical processing of somatosensory inputs.

Water immersion can alter numerous physiological parameters depending on physical characteristics like buoyancy, hydrostatic pressure, and temperature. Water immersion can provide relief from edema and improve blood flow [12,13], and these effects are beneficial for the rehabilitation of patients with orthopedic [14], cardiovascular [15], or respiratory disorders [16]. On the other hand, the effects of water immersion on somatosensory cortical processing have never been examined, despite the fact that water immersion is a form of multimodal somatosensory stimulation, engaging tactile-, pressure-, and thermosensitive pathways, and so would be expected to evoke widespread cortical activity. The present results provide a foundation for further studies on the benefits of aquatic rehabilitation for frail elderly and patients with neurological disorders, such as hemiplegic stroke patients.

Somatosensory inputs can attenuate short-latency SEPs evoked by other inputs, most often stimulation of the median nerve [5,7,9,10,17]. Jones et al. [8] demonstrated that interfering tactile stimuli, such as continuous rubbing of the palm ipsilateral to the median nerve, attenuated P25 and P29 due to centripetal gating. Acupuncture and tactile stimulation with a soft nylon brush to the ipsilateral palm also attenuated the parietal P22 (corresponding to P25) evoked by median nerve stimuli [18]. The author [18] suggested that the suppression of P22 is due to a uniform decrease in neuronal activity within the somatosensory area due to "afferent inhibition" [19].

We were uncertain why attenuation of the P25 component occurred in the present study under immersion conditions, since the hand that was innervated by the median nerve was not actually in the water. According to an earlier study [7] that examined changes in the waveform of SEPs due to continuous tactile stimuli to various parts of the body, including the face, hand, forearm, and foot both ipsilateral and contralateral to the median nerve stimuli, tactile stimuli of remote regions resulted in a consistent difference in the waveforms of short-latency SEPs. The authors proposed that this may be analogous to the phenomenon of "surround inhibition" described by Mountcastle and Powell [19] in which single unit responses were observed in the sensory cortex of the monkey. Additionally, they suggested that all areas of the skin may influence the cortical responses induced by median nerve stimuli. Therefore, somatosensory input from the whole of the body while immersed in water may have resulted in attenuation of the P25 component in the present study, but further studies are necessary to confirm this possibility and determine the underlying mechanisms.

The mapped P25 field is always confined to the contralateral parietal scalp and is thought to reflect activation in the various somatosensory receiving areas [20]. Indeed, patients with complete parietal vascular lesions and hemianesthesia lost both N20 and P25 [21]. Desmedt and Tomberg [22] examined the topographical patterns of short-latency SEPs and proposed that P27, which corresponds to P25 in the present study, reflects radially oriented neural generators in parietal area 1. Short-latency (20-40 ms) responses are generated mainly in areas 3b and 1 of the contralateral SI [23]. In a previous study that examined intracortical connectivity to the afferent input from skin in monkeys, area 1 was shown to receive a direct thalamocortical projection from the VPLc, as well as a corticocortical projection from area 3b [24]. According to a functional magnetic resonance imaging (fMRI) study, activity in areas 3 and 1 increased when pressure without pain was applied to the fingertip and antebrachial regions [25]. Therefore, the attenuation of P25 in the present study suggests that the somatosensory input from the immersed area of skin could alter excitability in areas 3b and 1.

The P45 field is inconsistent in young adults and its distribution over the scalp is quite variable. It always involves the contralateral central region, but can also extend over the front of the scalp [20]. This is in agreement with the present results, as 40-50 ms responses were found at all electrodes. Furthermore, P45 was recorded precentrally in a patient with complete destruction of the parietal cortex [21]; therefore, the P45 generator cannot be uniquely parietal.

The attenuation of P45 in the present study could be explained due to several cortical activities which were induced by the somatosensory input from water. Kawashima et al. [18] reported that attenuation of P40 (which corresponds to P45 in the present study) was induced by interfering tactile stimuli. They suggested that the generator of P40 is area 2 based on the dipole tracing method. However, others have proposed that the generator of P45 is located in SI [26]. As described above, although the generator of P45 is still unknown, it is possible that P45 reflects activity in the SI. Therefore, somatosensory input to the SI as the result of water immersion might induce the attenuation of P45.

An added possible explanation for the attenuation of P45 involves that there is an effect from activity in the PPC. Mauguiere et al. [21] proposed that P45 in healthy adults could reflect activity in several areas of the parietal cortex, including SI and the PPC. Inui et al. [27] investigated the temporal relationship among cortical responses to somatosensory stimuli using magnetoencephalography (MEG) and found that the activities peaking at 29 and 37 ms were located in the postcentral gyrus, corresponding to the PPC. Although a few MEG studies reported activation in this area at a latency of 50-100 ms following somatosensory stimuli [28,29], the main inputs to this area are from areas 1 and 2 in macaque monkeys [30]. Therefore, somatosensory input from water immersion to the PPC via SI might lead to the attenuation of P45. Furthermore, Inui et al. [27] proposed a hierarchical scheme of somatosensory processing from area 3b (peaking at 21-30 ms) to area 1 (peaking at 25-34 ms) and the PPC (peaking at 29-37 ms). Therefore, the attenuations of P25 (peaking at 23-30 ms) and P45 (peaking at 37-46 ms) in the present study might indicate a hierarchical processing from area SI to PPC of somatosensory input induced by water immersion.

The other possible explanation for the attenuation of P45 assumes that there is an effect from activity in the supplementary motor area (SMA) as well as the SI. The SMA does not appear to receive direct somatosensory input from the thalamus, but in monkeys it does receive projections from SI, SII, and area 5. Some neurons in the SMA respond to mechanical stimuli, such as light pressure or limb displacement [31]. Furthermore, Bodegard et al. [32] used fMRI in humans to show that light pressure to the index finger increased SMA activation. Additionally, a 40 ms positive potential was generated in a hand representation of the SMA [23]. Since afferent inputs are transmitted to the SMA via SI and the PPC [33], the present results cannot rule out this possibility.

## Conclusion

We demonstrated that water immersion modulates the short-latency SEPs known to originate in the SI. We propose that attenuation of short-latency SEPs is caused by somatosensory inputs from water, and that water immersion influences the cortical processing of other somatosensory inputs.

## Methods

### Subjects

Somatosensory-evoked potential (SEP) recordings were obtained in ten healthy male volunteers age 20-30 years (mean age, 21.8 ± 2.5 years) who gave their informed consent before the study commenced. All subjects were right-handed, none had a history of neurological or psychiatric disease, and none were taking any medications. The present study was performed in accordance with the Declaration of Helsinki and approved by the local ethical committee.

### SEPs recordings and stimulus

The subjects wore only swimwear and were seated on a comfortable reclining armchair with a mounted headrest surrounded by shielding curtain to prevent electrical interference. They were instructed to relax during the SEP measurements and to ignore the stimulus. A SynAmps amplifier system controlled by Scan 4.3 software (Neuroscan, El Paso, TX, USA) was used to record EEG data. A 32-electrode Neuroscan Quickcap™ based on the 10-20 system was positioned on the subject's head, and conducting gel was applied to each electrode to establish and maintain scalp contact. Recordings were obtained from nine scalp electrodes placed at F3/F4, C3/C4, P3/P4, Fz, Cz, and Pz. All of the scalp electrodes were referenced to linked earlobe electrodes. Electrode impedances were kept below 5 kΩ. The sampling rate was 1,000 Hz.

The right median nerve at the wrist was electrically stimulated with 0.2 ms pulses at 3 Hz using a conventional bipolar felt-tipped electrode. The intensity of the stimulus (7.4 ± 2.3 mA) was fixed at about three times the sensory threshold (mean, 2.5 ± 0.7 mA). Stimulus intensity was always below the pain threshold for each subject.

## Conditions

**Figure 2:**
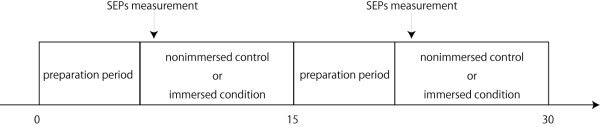
**The experimental procedure in the present study**. During the preparation period, we confirmed electrode impedances, water and ambient temperature, and body position of the subject.

**Figure 3:**
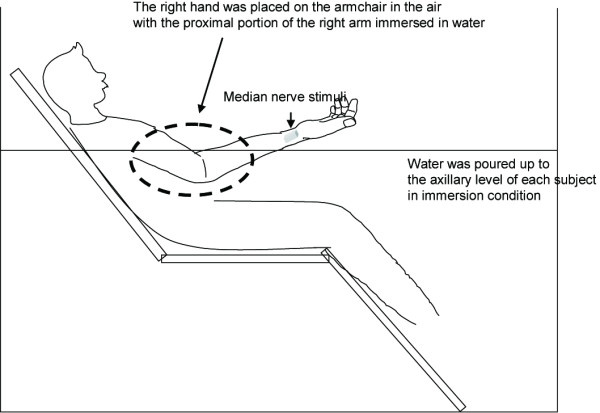
**The experimental setup used to measure SEPs under nonimmersed control and immersed conditions**.

The SEPs were measured while they were at rest both in and out of water. The subjects maintained the same body position under both immersed and nonimmersed control conditions. The entire experiment required 30 min, including the preparation time (Figure 2). Measurements under immersed conditions began immediately after water immersion. For the nonimmersed control measurements, the ambient temperature was set at 30°C, while for immersed SEP measurements, both ambient and water temperatures were set at 30°C. Water was poured up to the axillary level of each subject. The right hand was placed on the armchair above the water level with the proximal portion of the right arm immersed in water (Figure 3). To avoid carryover effects, SEP measurements under nonimmersed control and immersed conditions were performed in random order.

### SEP analysis

All data were stored in a personal computer, and signal processing software (NeuroScan) was used for analysis. The continuous EEG was segmented into epochs of 70 ms that included a 10 ms prestimulus period. Before averaging, each epoch underwent correction of slow linear trends by high-pass filtering and baseline correction using the prestimulus period. A high-pass filter (1 Hz, 6 dB/oct) was applied, together with a 50 Hz notch filter. Epochs were visually inspected and rejected from the average if voltage variations exceeded ± 70 μV. The remaining data (> 500 epochs) were averaged.

The amplitudes of P20, N30, and P45 measured at electrodes F3 and Fz, N18, P22, N30, and P45 measured at electrodes C3 and Cz, and N20, P25, N33, and P45 measured at electrodes P3 and Pz were identified on the basis of their latency, polarity, and scalp distribution. The peak amplitude of the SEPs was referenced to the peak of the preceding response. The peak amplitudes of the first components were defined from baseline-to-peak. The latencies were measured from stimulus onset to the peak of each component. The amplitudes and latencies of all components measured at all electrodes were analyzed by a two-factor repeated measures ANOVA for condition and electrode, and Tukey's post hoc tests were used for pair-wise comparisons. If the assumption of sphericity was violated in Mauchly's sphericity test, the degree of freedom was corrected using Greenhouse-Geisser's correction coefficient epsilon, and F- and P- values were recalculated. The significance level was set at 5%.

## List of abbreviations

SEP: somatosensory evoked potential; fMRI: functional magnetic resonance imaging; SI: primary somatosensory area; PPC: posterior parietal cortex; SMA: supplementary motor area.

## Authors' contributions

DS conceived of the experiment and was the primary investigator involved in the data collection and analysis as well as drafting of the manuscript. KY, HO, and AM (senior author) contributed to the experimental design, data analysis, and manuscript editing. YS contributed to manuscript editing. YT contributed to the data collection. All authors read and approved the final manuscript.
